# Editorial: The multidisciplinary focus on vascular access in patients with end-stage renal disease

**DOI:** 10.3389/fneph.2023.1244073

**Published:** 2023-08-14

**Authors:** Edwin Castillo Velarde

**Affiliations:** ^1^ INICIB, Instituto de Investigación de Ciencias Biomédicas, Universidad Ricardo Palma, Lima, Peru; ^2^ Hospital Guillermo Almenara, Lima, Peru

**Keywords:** vascular access (VA), hemodialysis, chronic renal disease (CKD), *Arteriovenous fístula*, catheter-related bloodstream infection (CRBSI)

Currently, our mission in vascular access care for patients with end-stage renal disease is multidisciplinary integration. In clinical practice, there is a dissociation between our theoretical knowledge and practice due to an individual or exclusive perspective between the specialties of vascular surgery, vascular interventional radiology, and nephrology. The most pragmatic and unfortunate example is the high prevalence of central venous catheter use in most countries and the heterogeneous prevalence with low incidence reports of arteriovenous native (AVFn) creation, despite the recommendation as first access from the guidelines for many years, even if there is an individualized focus (1, 2).

Nephrology has a leadership role in the care of patients with nephropathy and therefore has a responsibility to provide recommendations to improve the flow of vascular access management. In clinical practice, the interaction between the disciplines is still far from optimal. For example, the surgical vascular department may not be aware of the myriad of complications associated with the use of a central venous catheter, such as stenosis, thrombosis, infection, recurrent hospitalizations, and death, that are seen only in a nephrology service (3, 4). The vascular interventional radiology department has grasped the prevalence and incidence of complications such as stenosis and thrombosis; however, communication between all disciplines remains a goal to be pursued.

This Research Topic approaches a multidisciplinary focus on vascular access in patients with end-stage renal disease. Neyra et al. reviewed the natural history of vascular access. Many concepts of AVFs, catheters, and arteriovenous grafts are considered, such as patency rates, the process of fistula maturation, endovascular AVF creation, catheter design, the problem of bloodstream infections, catheter dysfunction, and the management of thoracic central vein obstruction (TCVO).

Contributions from Latin America have been included in this Research Topic to acknowledge the state of the art in vascular access. Portiolli et al. explored the availability of vascular access according to different healthcare systems in Brazil. It is important to analyze the different prevalences of non-tunneled catheters, tunneled catheters, and AVFs in countries with bipartite and tripartite health systems, which are extensive in many countries. In Peru, Castillo et al. dissected and compared the non-multidisciplinary perspective on vascular access with the integrative and multidisciplinary patient-centered perspective. The first fragmented perspective is a problem shared by many countries, and the consequences are higher costs, hospitalizations, and lower AVF creation because demand exceeds supply. The only way out is to change the paradigm with an integrative focus. It is significant the efforts from Peru to reach this objective. At the heart of this integration is the patient and their life project, which is stated by the KDOQI in its latest update (2). The patient’s preferences need to be taken into account in the decision-making process to properly plan vascular access. For example, with the introduction of ultrasound, proper vascular access to the AVF is required before the placement of a central venous catheter in the incident patient on dialysis. Right jugular access would not be recommended if the best option was an ipsilateral fistula. Patient empowerment is not only related to preserving their veins but also involves understanding concepts such as the flow of AVF or the need for Duplex ultrasound assessment (5).

Finally, regarding the need for an integrative focus, Garcia-Yañez et al. presented the problem of vascular access, supported by epidemiological data in Mexico. Again, demand exceeds supply. The low prevalence of AVF is the tip of the iceberg, and there is a need to analyze other variables like local practices and the health system. Beyond safeguarding a standard work policy, the decisions for the future of vascular access must focus on patient-centered care (5, 6).

## Author contributions

The author confirms being the sole contributor of this work and has approved it for publication.
